# Roles for the Conserved Spc105p/Kre28p Complex in Kinetochore-Microtubule Binding and the Spindle Assembly Checkpoint

**DOI:** 10.1371/journal.pone.0007640

**Published:** 2009-10-28

**Authors:** Cinzia Pagliuca, Viji M. Draviam, Eugenio Marco, Peter K. Sorger, Peter De Wulf

**Affiliations:** 1 Department of Experimental Oncology, European Institute of Oncology, Milan, Italy; 2 Biological Engineering Division, Massachusetts Institute of Technology, Cambridge, Massachusetts, United States of America; 3 Department of Systems Biology, Harvard Medical School, Boston, Massachusetts, United States of America; 4 Department of Genetics, University of Cambridge, Cambridge, United Kingdom; University of Edinburgh, United Kingdom

## Abstract

**Background:**

Kinetochores attach sister chromatids to microtubules of the mitotic spindle and orchestrate chromosome disjunction at anaphase. Although *S. cerevisiae* has the simplest known kinetochores, they nonetheless contain ∼70 subunits that assemble on centromeric DNA in a hierarchical manner. Developing an accurate picture of the DNA-binding, linker and microtubule-binding layers of kinetochores, including the functions of individual proteins in these layers, is a key challenge in the field of yeast chromosome segregation. Moreover, comparison of orthologous proteins in yeast and humans promises to extend insight obtained from the study of simple fungal kinetochores to complex animal cell kinetochores.

**Principal Findings:**

We show that *S. cerevisiae* Spc105p forms a heterotrimeric complex with Kre28p, the likely orthologue of the metazoan kinetochore protein Zwint-1. Through systematic analysis of interdependencies among kinetochore complexes, focused on Spc105p/Kre28p, we develop a comprehensive picture of the assembly hierarchy of budding yeast kinetochores. We find Spc105p/Kre28p to comprise the third linker complex that, along with the Ndc80 and MIND linker complexes, is responsible for bridging between centromeric heterochromatin and kinetochore MAPs and motors. Like the Ndc80 complex, Spc105p/Kre28p is also essential for kinetochore binding by components of the spindle assembly checkpoint. Moreover, these functions are conserved in human cells.

**Conclusions/Significance:**

Spc105p/Kre28p is the last of the core linker complexes to be analyzed in yeast and we show it to be required for kinetochore binding by a discrete subset of kMAPs (Bim1p, Bik1p, Slk19p) and motors (Cin8p, Kar3p), all of which are nonessential. Strikingly, dissociation of these proteins from kinetochores prevents bipolar attachment, even though the Ndc80 and DASH complexes, the two best-studied kMAPs, are still present. The failure of Spc105 deficient kinetochores to bind correctly to spindle microtubules and to recruit checkpoint proteins in yeast and human cells explains the observed severity of missegregation phenotypes.

## Introduction

Bipolar attachment of sister chromatids to spindle microtubules (MTs) depends on the correct assembly of kinetochores. Kinetochores contain ∼70 protein subunits in *S. cerevisiae* and >120 in humans. In *S. cerevisiae*, association of all known kinetochore proteins with centromeric (*CEN*) DNA requires prior binding of the four-protein CBF3 complex to a highly conserved *CEN* sequence, CDEIII. Additional DNA-binding, linker and MT-binding proteins are then recruited to *CEN*-bound CBF3. Assembly is hierarchical in that DNA-binding proteins require CBF3 for *CEN* association but not other kinetochore components; linker proteins require DNA-binding but not MT-binding subunits, and MT-binding subunits require both DNA-binding and linker proteins [1]–[3]. The composition and architecture of the MT-binding interface on kinetochores remains poorly defined but is known to comprise more than a dozen kMAPs and kinesins, most of which are present in multiple copies per kinetochore [4]. Moreover, several linker complexes (most notably the Ndc80 complex) also bind directly to MTs [5], [6]. Determining how the activities of this multiplicity of kMAPs and motors are coordinated to promote stable MT attachment, power poleward and anti-poleward movement, and spindle assembly checkpoint activity remains a major challenge.

Budding yeast Spc105p was identified as a protein that co-purifies with COMA complex subunit Mcm21p [7], and with Mtw1/MIND complex subunits Mtw1p, Nsl1p, and Dsn1p [7], [8]. Subsequent mass spectrometry of affinity purified Spc105p-PrA showed YDR532Cp (Kre28p) to be an associated protein [8]. Electron microscopy (EM), fluorescence imaging, and chromatin immunoprecipitation (ChIP) demonstrated that Spc105p and Kre28p are kinetochore components that localize to *CEN* DNA throughout the yeast cell cycle [7], [8]. Neither protein is present along MTs, at spindle pole bodies, or at the spindle midzone, locations at which other kinetochore components are found [9], [10].

In *C. elegans*, RNAi mediated depletion of Spc105p ortholog KNL-1 prevents kinetochore assembly and generates a kinetochore-null phenotype [11], [12]. In human cells, RNAi of hSPC105 (also known as AF15q14/CASC5 [13], D40 [14], Blinkin [15], and hKLN1 [16]) causes detachment of sister chromatids from the spindle despite continued presence on kinetochores of the hNDC80 complex; the primary factor in kinetochore-MT attachment [15]. In addition, the cells are spindle checkpoint defective [15]. In *Drosophila*, loss of Spc105 activity results in aberrant kinetochore formation and inability of the sister chromatids to align to the spindle. Importantly, the spindle checkpoint is proficient in *Drosophila* lacking Spc105 activity [17]. In fission yeast, the Spc105p ortholog named Spc7 is essential for kinetochore attachment and spindle integrity [18], [19]. In budding yeast, Spc105p is essential for viability [8]. However, whether this protein is part of a kinetochore subcomplex or acts as a monomer, how it is recruited to centromeres, whether it binds to MTs, contributes to spindle integrity or is required for the spindle checkpoint response in budding yeast is unknown. We show here that *S. cerevisiae* Spc105p and Kre28p form a 1∶2 heterotrimeric complex, which is recruited to centromeres only by the CBF3 complex. Its localization to kinetochores does not depend on any other DNA-binding component, nor on linker or MT-binding proteins/complexes. The Spc105 complex is highly elongated and binds to MTs, albeit with low affinity, establishing it as a novel budding yeast kinetochore linker unit that bridges centromeres and MTs. Spc105p is not required for MT binding *per se* but ensures bi-orientation of sister chromatids on the mitotic spindle by recruiting a discrete set of conserved kMAPs and kinesins. A second role of the Spc105p/Kre28p complex consists of maintaining the spindle assembly checkpoint -recruited by the conserved Ndc80 complex- to misaligned kinetochores allowing cells sufficient time to correctly orient their sister chromatids on the spindle structure. In human cells, we show that hSPC105 contributes similarly to the recruitment of certain kMAPS to the outer kinetochore, but that it is essential for kinetochore-MT binding. hSPC105 is required for correct mitotic timing and spindle checkpoint activity. We show that in human cells its contribution to the spindle checkpoint response is more important that that of the hNDC80 complex. The latter recruits a subset of checkpoint proteins (not including hBUB1 or hBUBR1), while all checkpoint proteins tested require hSPC105 for their localization to misaligned sister chromatids. A combination of incorrect MT attachment and spindle checkpoint defects explains the massive chromosome mis-segregation observed in budding yeast and human cells lacking Spc105 activity.

## Results

### Spc105p and Kre28p form an essential, conserved kinetochore complex

**Figure 1:**
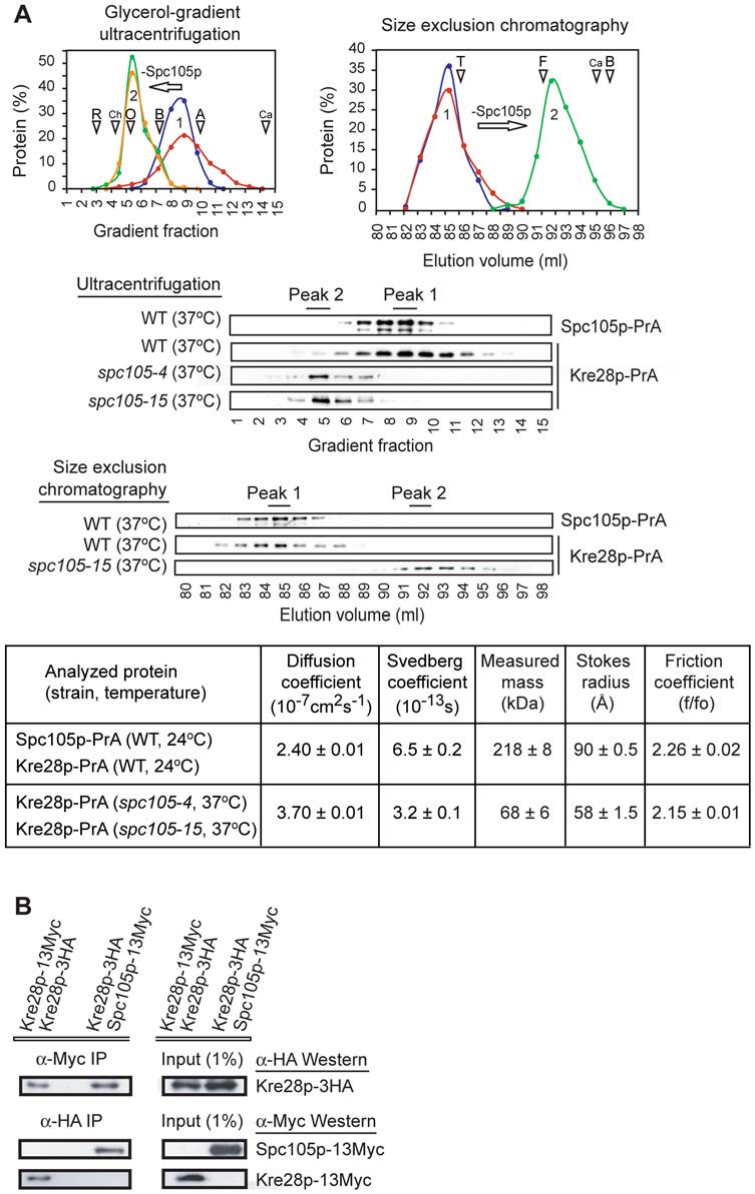
Biochemical characterization of the budding yeast Spc105p/Kre28p complex. **(*A*)** Hydrodynamic analysis of whole cell extracts prepared from wild-type (WT), *spc105-4*, and *spc105-15* strains expressing ectopic Spc105p-PrA or Kre28p-PrA. Each strain was grown at 24°C and then shifted to 37°C (3 h) prior to preparation of cell extracts. Proteins were detected by western blotting with anti-PrA antibodies. Triangles indicate the positions of standard proteins (R: ribonuclease A, Ch: chymotrypsinogen, O: ovalbumin, B: bovine serum albumin, A: aldolase, Ca: Catalase, T: thyroglobulin, F: ferritin). **(*B*)** Co-immunoprecipitation of Kre28p and Spc105p from whole cell extracts prepared from diploid *KRE28::13Myc KRE28::3HA* and *KRE28::3HA SPC105::13Myc* strains.

We have used simple hydrodynamic analysis to determine the assembly state of budding yeast Spc105p, following procedures we have applied previously to other kinetochore substructures [7]. Chromosomal copies of *SPC105* and *KRE28* were fused at their 3′ ends to Protein A (PrA) and cell extracts expressing the fusion proteins were analyzed by sedimentation velocity ultracentrifugation on 10-40% glycerol gradients and by size exclusion chromatography on Sephacryl HR-500 columns. Fractions containing Spc105p-PrA or Kre28p-PrA were identified by western blotting and elution volumes compared to those of standards with known diffusion and sedimentation coefficients (Fig. 1A). Spc105p-PrA and Kre28p-PrA were found to co-migrate on columns and gradients and to have hydrodynamic properties consistent with an elongated ∼218 kDa complex (diffusion coefficient of 2.4×10^-7^ cm^2^s^−1^, sedimentation coefficient of 6.5×10^−13^ s, frictional coefficient >2.0; Fig. 1A). Other yeast kinetochore proteins or complexes, including Stu2p, Kar3p, and the Ndc80 complex have similarly high frictional coefficients [7], [20]–[22] and have been shown by EM to be rod-like [20]–[22].

Tight binding of Spc105p to Kre28p in native complexes was confirmed by reciprocal immunoprecipitation (IP) from extracts of diploid strains ectopically expressing Kre28p-3HA and Spc105p-13Myc. Anti-HA antibodies pulled down Spc105p-13Myc, while anti-Myc antibodies pulled down Kre28p-3HA from *KRE28::3HA SPC105::13Myc* cell extracts (Fig. 1B). Given subunit masses of 105 kDa for Spc105p and 45 kDa for Kre28p, the apparent native molecular weight of the Spc105p-PrA complex is larger than expected for a 1:1 assembly (even accounting for a 15 kDa PrA tag), suggesting that more than one Kre28p subunit is present. In support of this hypothesis, we found Kre28p-3HA and Kre28p-13Myc to co-IP from extracts made from *KRE28::3HA KRE28::13Myc* cells (Fig. 1B). In extracts from *KRE28::PrA spc105-4 or KRE28::PrA spc105-15* mutant cells, shifted for 3.5 h to non-permissive conditions (37°C), the measured mass of Kre28p-PrA was that of a PrA-tagged monomer: ∼68 kDa (Fig. 1A). Together, these results show that the Spc105p/Kre28p complex contains two Kre28p subunits that bind independently to Spc105p (rather than to each other), perhaps via the coiled-coils present in both proteins.

**Figure 2:**
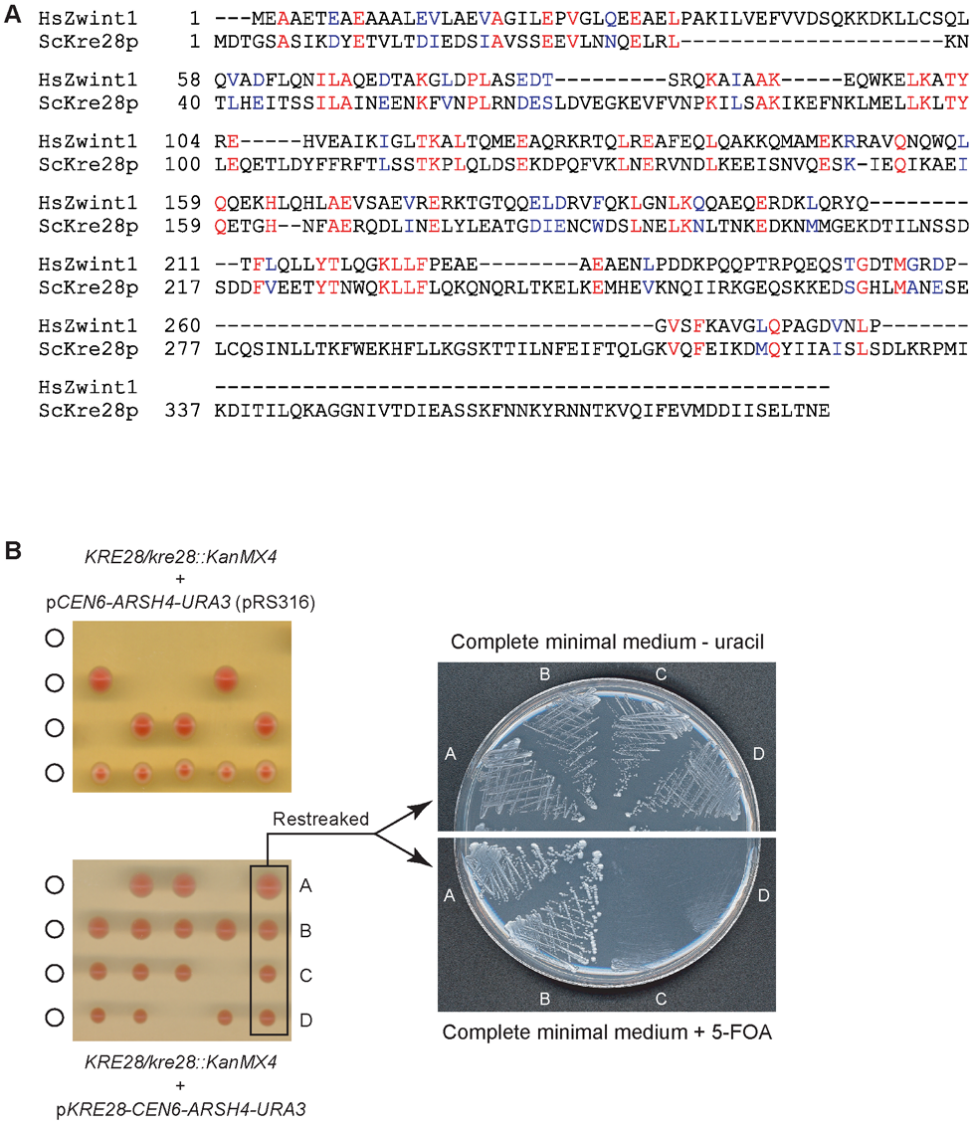
Kre28p, the candidate orthologue of metazoan Zwint-1, is essential for the viability of budding yeast. **(*A*)** Protein sequence alignment (T-Coffee) of human Zwint-1 and *Saccharomyces cerevisiae* Kre28p. Residues labeled red are identical, residues labeled blue are similar. **(*B*)** *KRE28* is essential for the viability of *S. cerevisiae* W303 as determined by gene complementation and tetrad analysis (see text for details).

Four pieces of data suggest that Kre28p is likely to be the orthologue of metazoan kinetochore protein Zwint-1: (i) Zwint-1 and Kre28p exhibit fair sequence conservation (Fig. 2A), (ii) Zwint-1 and SPC105 co-purify from extracts of chicken DT40 cells (Fukagawa T., personal communication), (iii) human Zwint-1 and hSPC105 interact in two-hybrid assays [15], and (iv) localization of Zwint-1 to human kinetochores depends on hSPC105 [15], [16]. Future biochemical analysis will be required to show that human Zwint-1 and hSPC105 form a heterotrimer.

Although listed as non-essential in the *S. cerevisiae* genome database (http://www.yeastgenome.org/cgi-bin/locus.fpl?locus=kre28) we found *KRE28* to be essential in a W303 background. When a diploid strain heterozygous for a *kre28::KanMX4* disruption was sporulated, a kan***^−^*** phenotype segregated 2:0 (Fig. 2B). Introduction of a *URA3 CEN* plasmid expressing *KRE28* from its own promoter generated tetrads in which the *kan* ***^+^*** *kre28Δ* spores were 5-fluoroorotic acid (5-FOA) sensitive, while the *kan* ***^−^*** *KRE28* spores were 5-FOA resistant. These results demonstrate that *KRE28*, like *SPC105*, is essential for vegetative growth of W303 strains. We have not yet determined whether *KRE28* is dispensable in the S288C background used by the genome database or whether the annotation as “non-essential” is incorrect. However, we have observed papillae when W303 *kan* ***^+^*** *URA3 CEN* strains are transferred to 5-FOA, implying relatively frequent emergence of suppressors.

### Binding of the Spc105p/Kre28p complex to kinetochores requires only CBF3

To determine which kinetochore proteins are required for *CEN*-binding by Spc105p/Kre28p, we used chromatin immunopreciptation (ChIP) to follow crosslinking of Spc105p-GFP and Kre28p-GFP to *CEN4* in a series of congenic strains carry carrying temperature-sensitive mutations in kinetochore subunits. To monitor the efficiency of temperature-dependent protein inactions, we performed in parallel positive and negative control experiments involving known interdependencies (Table 1, Fig. S1). *CEN4* binding by Spc105p/Kre28p was eliminated by *ndc10-1, w*hich inactivates CBF3, but not by mutations in two other DNA-binding kinetochore subunits: *mif2-3* and *cse4-1*. Binding by several proteins to kinetochores is disrupted in *mif2-3* and *cse4-1* cells (e.g., the COMA and MIND complexes [23]-[25]). Because *cse4-1* is a hypomorphic mutation [26], *CEN4* binding by Spc105p/Kre28p in *cse4-1* cells does not demonstrate that centromeric chromatin is truly dispensable, but it does distinguish the Spc105p/Kre28p complex from COMA and MIND. We observed kinetochore recruitment of Spc105p/Kre28p to be retained in cells carrying mutations in the MIND, COMA or Ndc80 linker complexes and in these cases, control experiments demonstrated effective inactivation of the mutant proteins (Table 1, Fig. S1). We therefore conclude that assembly of Spc105p/Kre28p on kinetochores depends on CBF3 but not on other DNA-binding or linker complexes.

**Table 1:** *CEN*-recruitment of kineotochore proteins in yeast kinetochore mutants.

		Spc105p-GFP		Cep3p		Negative control		
Complex	Mutant	24°C	37°C	24°C	37°C		24°C	37°C
CBF3	*ndc10-1*	+	-	+	−			
Cse4 nucleosome	*cse4-1*	+	+	+	+	Mif2p	+	±
Mif2p	*mif2-3*	+	+	+	+	Bim1p-GFP	+	−
COMA	*okp1-5*	+	+	+	+	Ame1p-TAP	+	−
MIND	*mtw1-1*	+	+	+	+	Bim1p-GFP	+	−
Ndc80	*ndc80-1*	+	+	+	+	Slk19p-GFP	+	−
	*spc25-7*	+	+	+	+	Ndc80p-GFP	+	−
						Cep3p		
Complex	Mutant	Analyzed protein		24°C	37°C	24°C	37°C	
Spc105	*spc105^ts^*	Kre28p-GFP		+	−	+	+	
		Spc105-4p-PrA		+	−	+	+	
		Spc105-15p-PrA		+	−	+	+	
CBF3	*spc105^ts^*	Cep3p		+	+	+	+	
Mif2p	*spc105^ts^*	Mif2p		+	+	+	+	
COMA	*spc105^ts^*	Okp1p-GFP		+	+	+	+	
MIND	*spc105^ts^*	Mtw1p-GFP		+	+	+	+	
Ndc80	*spc105^ts^*	Ndc80p-GFP		+	+	+	+	
		Spc25p-GFP		+	+	+	+	
		*spc105^ts^*			*ndc80-1*		*mtw1-1*	
Complex	Protein	24°C		37°C	24°C	37°C	24°C	37°C
CBF3	Cep3p	+		+	+	+	+	+
Bim1p	Bim1p-GFP	+		−	+	−	+	−
Bik1p	Bik1p-GFP	+		−	+	−	+	−
Kar3p	Kar3p-GFP	+		−	+	−	+	−
Slk19p	Slk19p-GFP	+		−	+	−	+	−
Cin8p	Cin8p-GFP	+		−	+^◊^	±^◊^	+	−
Kip1p	Kip1p-GFP	+		+	+	±	+	−
Kip3p	Kip3p-GFP	+		+	+	±	+	−
DASH	Spc19p-GFP	+		+	+**^#^**	−**^#^**	+	−
	Spc34p-PrA	N.D.		N.D.	N.D.	N.D.	+	−
Stu2p	Stu2p-GFP	+		+	+**^#^**	−**^#^**	+	−

*spc105^ts^*: *spc105-4* and *spc105-15*. ^◊^ [7], **^#^** [34]. N.D.: not determined. The ChIP data are shown in Fig. S1 and are sketched in Fig. 7A,B.

Next we examined the role played by Spc105p/Kre28p in *CEN4* binding by other kinetochore DNA-binding and linker proteins. We showed by ChIP that Cse4p, Mif2p and proteins in the COMA, MIND, and Ndc80 complexes crosslink to *CEN4* at 37°C with equal efficiency in *spc105-4*, *spc105-15* and wild-type cells. However, the *spc105-4* or *spc105-15* gene products were clearly temperature-sensitive for function: when either allele was linked to a 3′ PrA tag, *CEN4* crosslinking was lost at 37°C (Table 1, Fig. S1). Moreover, *CEN4* crosslinking by Kre28p-GFP was lost in *spc105-4* or *spc105-15* cells at 37°C. Thus, the DNA-binding Cse4p and Mif2p, and the linker COMA, MIND, and Ndc80 complexes can associate with *CEN* DNA in the absence of Spc105p/Kre28p function.

### The Spc105p/Kre28p complex recruits a subset of kMAPs and kinesins to kinetochores

Yeast kinetochores contain multiple motors and kMAPs; the relationships among these proteins and their interactions with linker proteins have not yet been explored systematically. We therefore examined the ability of nine known kMAPs and kinesins to crosslink to *CEN4* DNA in the presence of mutations in the Spc105 complex (*spc105-4* and *spc105-15*), the Ndc80 complex (*ndc80-1*) or the MIND complex (*mtw1-1*; Table 1, Fig. S1). In *spc105-4* and *spc105-15* mutants, the kMAPs Bim1p, Bik1p, and Slk19p, and kinesins Cin8p and Kar3p were lost from kinetochores whereas proteins such as Stu2p, Kip1p, Kip3p, and the Dam1/DDD/DASH complex were still bound (Table 1, Fig. S1). *CEN4* crosslinking by most kMAPs and kinesins was eliminated in *ndc80-1* cells but crosslinking to *CEN4* by Cin8p, Kip1p, and Kip3p was detectable at approximately 50% of wild-type levels. Finally, MIND was required for *CEN4* binding by all kinetochore kMAPs and motors assayed. These data demonstrate a requirement for the Spc105p/Kre28p, MIND and Ndc80 complexes in recruitment of kMAPs and motors to the outer kinetochore but show that no simple correspondence exists between linkers and kMAPs/motors; most if not all MT-binding kinetochore proteins appear to require more than one linker for *CEN* association.

### Spc105p binds to microtubules *in vitro*

**Figure 3:**
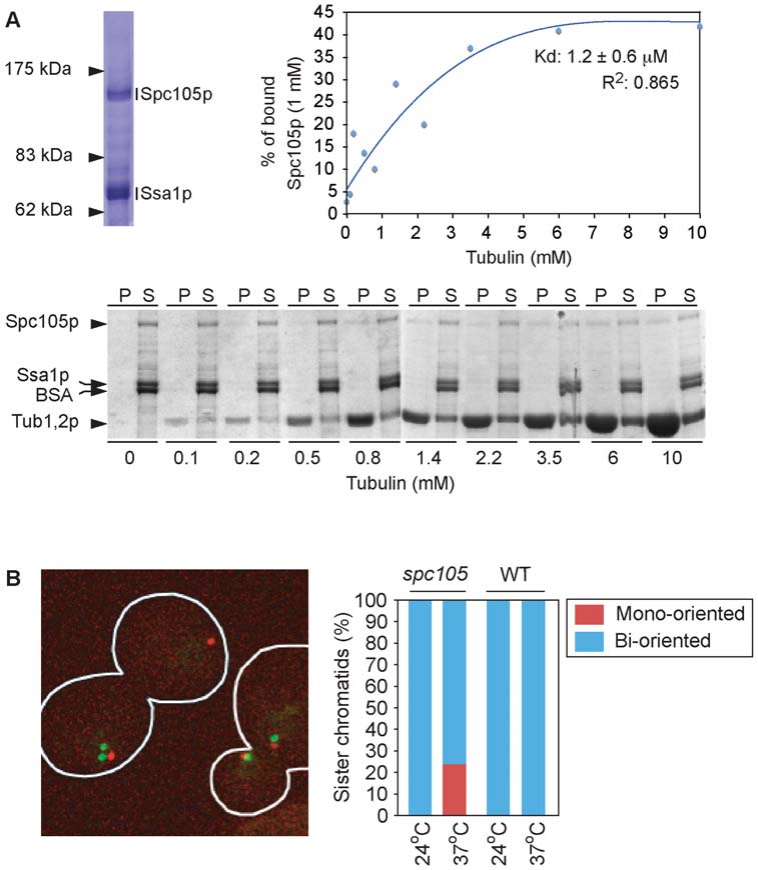
Budding yeast Spc105p binds to microtubules and is required for sister chromatid bi-orientation. **(*A*)** Affinity-purified Spc105p (Ssa1p is a co-purifying chaperon) binds to taxol-stabilized bovine MTs with a Kd of ∼1.2 µM as measured in MT-spindown experiments. **(*B*)** Loss of Spc105p/Kre28p activity increases the frequency of mono-oriented sister chromatids. In the microscopic image, the left *spc105* cell shows a mono-oriented sister chromatid 3 pair (green dots), while in the right cell, the sister chromatid 3 pair is bi-oriented on the spindle. Position of the spindle is indicated by the two spindle pole bodies colored red.

In addition to recruiting kMAPs and motors to MTs, the budding yeast Ndc80 kinetochore linker complex binds directly to MTs *in vitro* [6]. The *C. elegan*s Spc105 ortholog KNL-1 has also been shown to bind to MTs *in vitro* [27]. To determine whether this is true of *S. cerevisiae* Spc105p, we N-terminally fused *SPC105* to GST under control of the *GAL1* promoter and isolated the fusion protein from whole yeast cell extracts using glutathione beads (Fig. 3A). The beads were washed extensively and bound protein then incubated with Precision protease, releasing species of ∼70 kDa and ∼150 kDa (by SDS PAGE). Mass spectrometry confirmed the proteins to be chaperone Ssa1p and Spc105p, respectively. Kre28p was not present at detectable levels in column eluates, presumably because it was lost when the glutathione beads were washed. When purified Spc105p was added to taxol-stabilized bovine brain MTs, we observed MT binding with an apparent dissociation constant (Kd) of ∼1.2 µM±0.6 (Fig. 3A). The affinity of yeast Spc105p to MTs is relatively low compared to that of the DASH complex (Kd: 0.2 µM [28]), Stu2p (0.5 µM [29]), the Ndc80 complex (0.2–0.8 µM [6]) or Kar3p (0.7 µM [30]) but is similar to that observed for *C. elegans* KNL-1 (1-2 µM; Cheeseman IM, personal communication). To determine the functional significance of Spc105p binding to MTs it will be necessary introduce specific disrupting mutations into the putative MT-binding interface, a task that should be aided by ongoing crystallographic studies. Overall we conclude that the Spc105p/Kre28p complex plays an essential role in recruiting multiple kMAPs and motors to the outer kinetochore and may have an additional function in direct MT binding.

### Increased mono-orientation of sister chromatids in cells lacking Spc105p activity

To study the contributions of Spc105p/Kre28p to kinetochore-MT attachment we filmed *spc105* cells carrying a *CEN3* proximal chromosome tag. Binding of TetR-GFP to tandem TetO operator sites in the tag creates a bright fluorescent dot that can be tracked in 3D using machine vision software [31], [32]. We observed that sister chromatids in *spc105* cells did not bi-orient correctly on the mitotic spindle; ∼25% remained mono-oriented after 3h at 37° as compared to 0% at permissive temperature (Fig. 3B). In addition, among the subset of *spc105* sisters that managed to bi-orient, motion on the spindle was different from that observed in wild-type cells: a particularly dramatic reduction in away-from-the-pole velocity was observed (4.50±0.12 mm/min in wild-type at 37°C versus 3.90±0.15 mm/min in *spc105-4* at 37°C). Whereas strains mutant in the fission yeast *SPC105* ortholog *SPC7* have been reported to contain broken spindles [19] we did not observe any structural abnormalities in *spc105* spindles. Kinetochore mutations can cause spindle defects in budding yeast, as illustrated by mutations in the DASH complex [33], or in complexes required for the localization of DASH to kinetochores (COMA, MIND or the Ndc80 complexes [34], [35]; Table 1, Fig. S1), or in proteins involved in DASH regulation (e.g., the Ipl1 kinase [35]–[37]). One explanation for the absence of spindle defects in *spc105* budding yeast as compared to *spc7* fission yeast mutants is that kinetochore binding by MIND, DASH and other proteins are lost in the latter case, but not in the former [3].

### The Spc105p/Kre28p complex maintains the spindle assembly checkpoint at budding yeast kinetochores

The spindle assembly checkpoint inhibits activation of the anaphase-promoting complex when unattached or mal-oriented chromosomes are present. As a result, cells do not exit from mitosis, providing additional time for sister chromatids to achieve bi-orientation on the mitotic spindle [38]. Many kinetochore mutants provoke a strong checkpoint arrest (e.g., *cse4-1, scm3-1, mif2-3, ame1-4, okp1-5, nnf1-17, mtw1-1, stu2-277, dam1-1*) but a subset does not (e.g., *ndc10-1*, *cep3-1*, *spc25-7, ndc80-1*) despite severe defects in chromosome-MT binding. This arises because binding of the Mad and Bub spindle checkpoint proteins to unattached kinetochores, an essential step in checkpoint signaling, requires functional CBF3 and Ndc80 complexes [39]–[42]. It has been reported that COMA plays a similar role [43] but this seems unlikely to us since mutations in essential COMA subunits such as *ame1-4* and *okp1-5* provoke a strong mitotic arrest under non-permissive conditions [7], [43]. When *spc105-4* or *spc105-15* cells were shifted to 37°C they failed to arrest efficiently and exited from mitosis despite the presence of mono-oriented sister chromatids. The cells also failed to complete cytokinesis and gradually lost viability, phenotypes shared with *ndc10* and *ndc80* mutants (Fig. S2, [39]–[41]).

**Figure 4:**
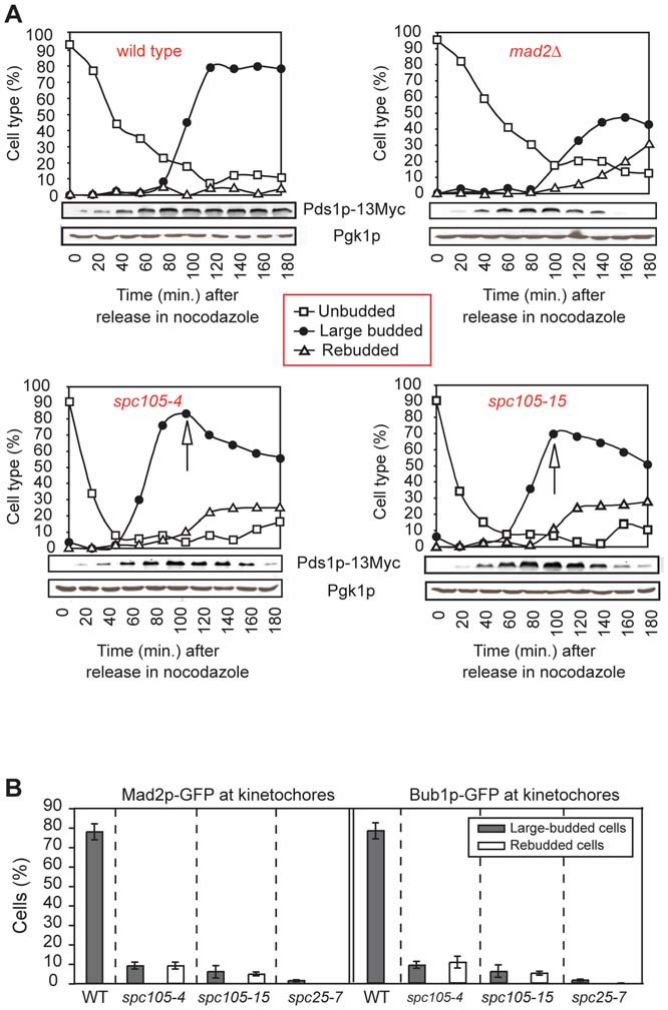
The Spc105 complex is essential for spindle checkpoint activity in yeast. **(*A*)** Wild-type (positive control), *mad2Δ* (negative control), *spc105-4,* and *spc105-15* cells were synchronized with α-factor and released into nocodazole-containing YPD at 37°C. Samples were analyzed by western blotting of Pds1p-13Myc with anti-Myc antibodies, and of Pgk1p (loading control) with anti-Pgk1p antibodies. Quantification of the budding index is also shown. The arrows show where exit from the SAC-mediated delay occurs. The flow cytometric data of DNA content are shown in Fig. S3. **(*B*)** Quantification of Mad2p-GFP and Bub1p-GFP recruitment to kinetochores in wild-type (positive control), *spc105-4*, and *spc105-15* cells (n = 100–200) synchronized with α-factor and released in nocodazole-containing YPD at 37°C. Similarly treated *spc25-7* cells, which lack a functional Ndc80 complex, serve as control for loss of Mad2p-GFP and Bub1p-GFP at kinetochores.

To assay spindle checkpoint function in *spc105-4* and *spc105-15* cells, they were synchronized in G1 with α-factor and released at 37°C into media containing MT poison nocodazole. Cell cycle progression was monitored by DNA content (FACS) and the levels of Pds1p-13Myc, a securin whose degradation at the metaphase-anaphase transition is required for sister chromatid separation (Fig. 4A, Fig. S3). Nocodazole-treated wild-type cells arrested with 2N DNA content and high Pds1p-13Myc levels, while *mad2Δ* cells, included as a control, proceeded through the metaphase-anaphase transition without delay, as evidenced by rapid Pds1p-13Myc destruction and the appearance of 4N cells. The timing of Pds1p-13Myc degradation in *spc105-4* and *spc105-15* was similar to that of *mad2Δ* cells, demonstrating a failure to arrest. When cell morphology was examined, transient accumulation of large-budded cells, which is diagnostic of G2/M cells, was observed in *spc105-4* and *spc105-15* but not in *mad2Δ* cells (Fig. 4A). These data suggest that *spc105-4* and *spc105-15* respond transiently to nocodazole. This conclusion is consistent with evidence of transient binding by Mad2p-GFP and Bub1p-GFP to kinetochores: at peak levels, only 5-10% of *spc105-4* and *spc105-15* cells contained kinetochore-bound Mad2p-GFP and Bub1p-GFP in contrast to >80% of wild-type cells (Fig. 4B). Mad2p-GFP and Bub1p-GFP binding to kinetochores was nonetheless 3-fold higher in *spc105-4* and *spc105-15* cells under non-permissive conditions, than in *spc25-7* cells, in which the Ndc80 complex is disrupted and the checkpoint completely inactive [42] (Fig. 4B). In some cases, partial loss of kinetochore binding by Mad2 and Bub1 reflects the presence of a hypomorphic allele in a kinetochore linker (e.g., *nuf2-61* [42]), but we think this is unlikely to be the case with *spc105-4* and *spc105-15* alleles because both mutations abolish Spc105p-*CEN* crosslinking (Table 1, Fig. S1). Instead, we hypothesize that the Ndc80 complex is required to recruit checkpoint proteins to kinetochores and that Spc105p/Kre28p is needed to stabilize this association when mal-oriented or unattached chromosomes are present.

### Comparing the functions of Spc105 in yeast and human cells

**Figure 5:**
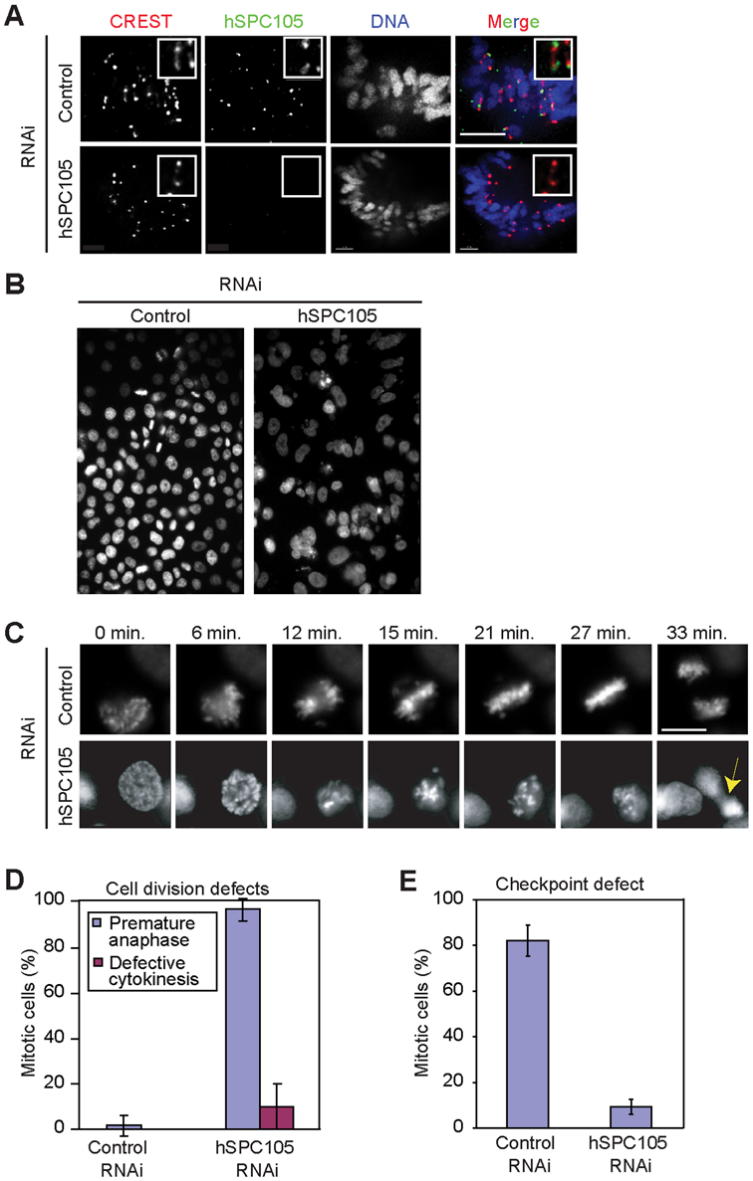
Human hSPC105 is essential for chromosome segregation, mitotic timing, cytokinesis, and spindle checkpoint activity. **(*A*)** Immunofluorescence imaging of mitotic HeLa cells following treatment with control or targeting siRNA oligos. Cells were stained with anti-hSPC105 antibodies (green), CREST sera (red) and DAPI (DNA, blue). Scale Bar: 10 µm. Insets are 13× magnified. **(*B*)** DAPI stained mitotic HeLa cells treated with hSPC105 targeting or control siRNA oligos. **(*C*)** Time-lapse imaging of histone 2B-GFP-expressing HeLa cells treated with hSPC105 targeting or control siRNA oligos. Images were collected every 3 min from ∼35 cells; representative frames are shown. In control cells (upper lane) anaphase occurs only after sister chromatid congression is complete, while in hSPC105-depleted cells (lower lane) anaphase is initiated in the presence of unaligned sisters, generating chromosome bridges (yellow arrow). **(*D*)** Cell division defects quantified from live-cell imaging experiments. **(*E*)** Percentage of nocodazole-treated control and hSPC105-depleted HeLa cells arresting in mitosis (as judged morphologically by DIC microscopy).

Are the Spc105p functions that we observed in yeast also conserved to humans? To address this question we first raised a polyclonal antibody against residues 1618–1634 of hSPC105. The antibody recognized a single protein on western blots with a mass of ∼300 kDa, as expected for hSPC105 (Fig. S4A), and also stained HeLa cell kinetochores throughout mitosis (Fig. 5A, Fig. S4B). RNAi against hSPC105 eliminated immune-reactivity by blotting and immunofluorescence imaging, demonstrating the specificity of the antibody (Fig. 5A,B; Fig. S4C).

**Figure 6:**
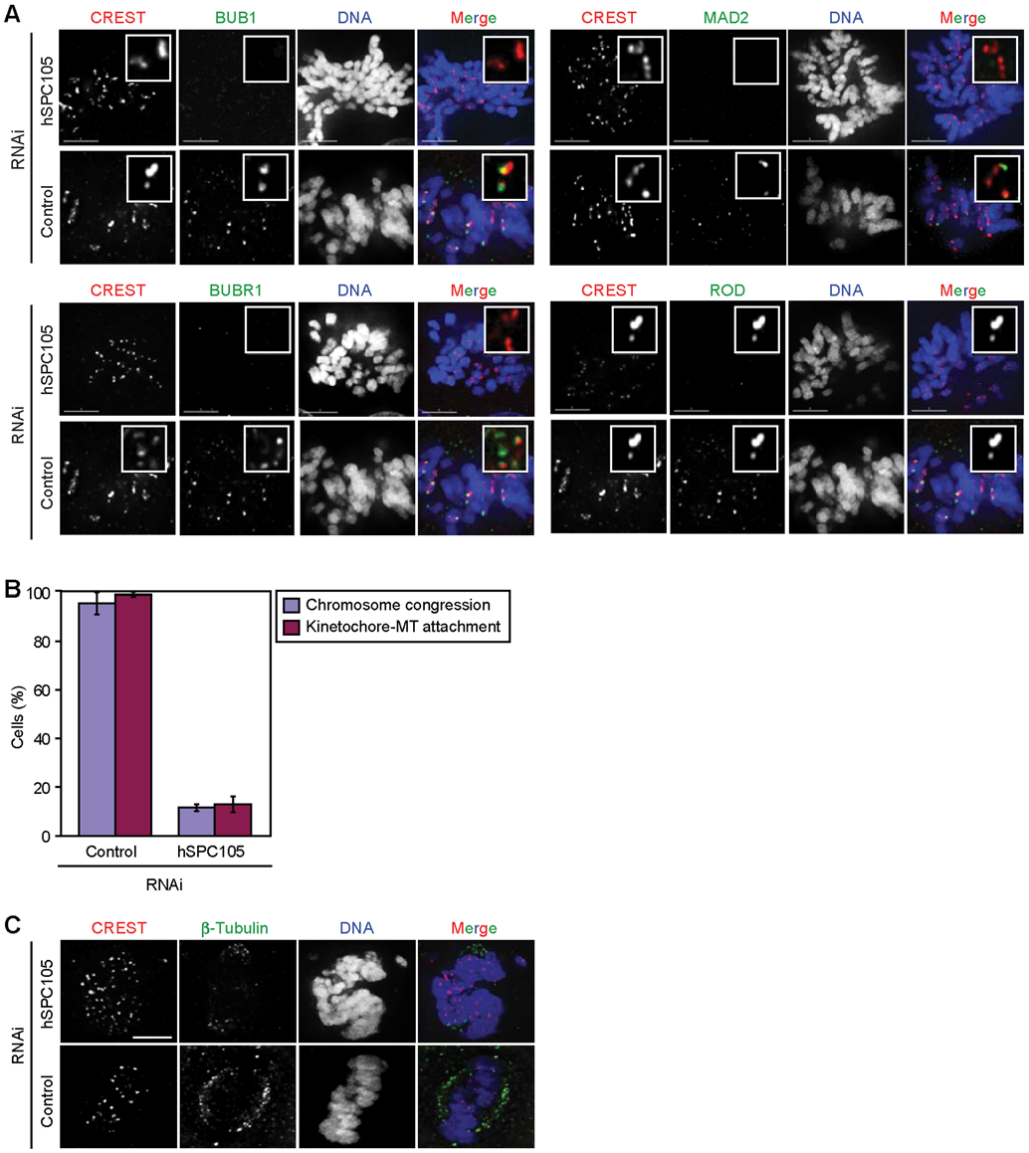
Human hSPC105 is essential for spindle checkpoint recruitment, kinetochore-MT attachment and chromosome congression. **(*A*)** Immunofluorescence imaging of hSPC105-depeted and control HeLa cells stained with CREST sera (inner kinetochore, red), DAPI (DNA, blue) and antibodies against hBUB1, hBUBR1, hMAD2 or ROD (green). ROD acts as a negative control. Scale Bar: 10 µm. Insets are 13× magnifications. **(*B*)** HeLa cells were treated with hSPC105 targeting or control siRNAs for 72 h, exposed to MG132 for 1 h (to block cells at the metaphase-anaphase transition), and subsequently cold-treated, fixed and stained. Percentage of hSPC105-depleted and control mitotic HeLa cells with congressed chromosomes (blue) and MT-attached kinetochores (red) were scored. **(*C*)** Immuno-fluorescence imaging of hSPC105-depleted and control mitotic HeLa cells stained with CREST sera (inner kinetochore, red), anti-β-tubulin antibodies (MTs, green), DAPI (DNA, blue). The status of chromosome congression, bipolar spindle formation and kinetochore-MT attachment are shown. Scale bar: 10 µm.

Human cells lacking hSPC105 activity are spindle checkpoint defective [15]. Following RNAi of hSPC105 in mitotic HeLa cells, we observed lagging sister chromatids in metaphase and widespread chromosome missegregation at anaphase (Fig. 5C, yellow arrow; Fig. 5D). The failure of hSPC105-depleted cells to arrest in the presence of mal-oriented chromosomes indeed suggests a defect in the checkpoint response. In support of this observation and that made by Kiyomitsu and colleagues [15], the robust mitotic arrest provoked in control cells by nocodazole was abolished in hSPC105-depleted cells (Fig. 5E). Lack of hSPC105 was shown to reduce the presence of spindle checkpoint proteins hBUB1 and hBUBR1 at human kinetochores [15]. We confirmed these findings and further showed that hMAD2 was absent from kinetochores in hSPC105-depleted HeLa cells, whereas all three proteins exhibited their normal pattern of kinetochore association on uncongressed chromosomes in control cells (Fig. 6A, Table 2). In budding yeast, Spc105p maintains the spindle checkpoint components -recruited by the Ndc80 complex- to misaligned sister kinetochores. Does hSPC105 mediate a similar supporting role in human cells? To investigate this possibility, we depleted hNDC80, which also disrupts the spindle checkpoint, and evaluated via immunofluorescence imaging the presence of hMAD2, hBUB1 and hBUBR1 at kinetochores. In contrast to our findings with budding yeast, lack of hNDC80 activity resulted in a loss of hMAD2 but not of hBUB1 and hBUBR1 from mal-oriented kinetochores (Table 2). Taken together, our data show that hSPC105 is actively involved in the recruitment of hMAD2, hBUB1 and hBUBR1 to human kinetochores, while the hNDC80 complex recruits only hMAD2. Thus, involvement of Spc105p in the spindle checkpoint response has evolved from a supporting role in stabilizing the checkpoint in budding yeast toward a most prominent role of recruiting and maintaining all spindle checkpoint components at human kinetochores.

**Table 2:** *CEN*-recruitment of proteins in HeLa cells lacking hNDC80 or hSPC105.

Analyzed protein	Control cells (no siRNA)	hNDC80 siRNA	hSPC105 siRNA
CREST (inner kinetochore marker)	+	+	+
hNDC80	+	−	+
hSPC105	+	+	−
EB1	+	+	−
p150^Glued^	+	−	−
CLIP-170	+	−	−
hBUB1	+	+	−
hBUBR1	+	+	−
hMAD2	+	−	−

(+): protein localizes to kinetochores, (−): protein does not localize to kinetochores.

During our experiments we observed that hSPC105-depleted cells fail to undergo chromosome congression (Fig. 6B). This phenotype may reflect a defect in the mechanics of kinetochore-MT binding or involvement of accelerated progress through mitosis [15]. We observed similar defects in chromosome congression in hSPC105-depleted cells in the presence and absence of proteosome inhibitor MG132, which blocks cells in anaphase (Fig. 6C) demonstrating that hSPC105-depleted kinetochores are unable to congress even when metaphase is greatly lengthened. Moreover, when hSPC105-depleted MG132-treated cells were briefly chilled, a treatment that depolymerizes MTs with free plus-ends, we observed significantly fewer MTs on hSPC105-depleted kinetochores than in control cells (Fig. 6B,C), confirming a role of hSPC105 in the formation of stable kinetochore-MT attachments [15].

Does hSPC105 depletion disrupt the recruitment of kMAPs and kinesins to outer kinetochores as observed in *spc105* budding yeast mutants? To address this question we examined hSPC105 RNAi-treated cells for kinetochore binding of a set of outer kinetochore components including EB1, dynein-dynactin (p150^Glued^), and CLIP-170 [44], [45] and of inner kinetochore components using CREST (because CREST staining did not vary across depletion conditions, it also served as control for the efficiency of fixation in different samples). Kinetochores of hSPC105-depleted cells showed strongly reduced levels of CLIP-170/Bik1p, EB1, and the dynein-dynactin motor complex (Table 2). Of note, in hNDC80-depleted cells we found that EB1 was still present at normal levels at outer kinetochores (Table 2). Thus, like its yeast counterpart, hSPC105 is involved in the recruitment of a discrete set of kMAPs and motors, further including CENP-F [16].

When the hSPC105 RNAi-treated cells were stained for hNDC80, we found that hSPC105 does not play a role in recruiting hNDC80, confirming previous observations [15], [16] However, does hSPC105 require hNDC80 for its localization to kinetochores? We found that elimination of hNDC80 did not affect kinetochore recruitment of hSPC105 (Table 2), confirming the recent finding that hSPC105 still localizes to kinetochores in absence of the hNDC80 complex [16]. We hence conclude that, similar to budding yeast, hSPC105 and hNDC80 are linker complexes that associate with kinetochores independently of each other. Presence of hNDC80 at outer kinetochores confirms that loss of hSPC105 does not interfere with kinetochore assembly or integrity - similar to our observations made with budding yeast.

## Discussion

In this paper we set out to study how conserved kinetochore protein Spc105p exists in, localizes to, and acts at kinetochores during mitosis in budding yeast, and whether these activities are conserved to humans. In *S. cerevisiae*, Spc105p forms a stable 1∶2 heterotrimeric complex with Kre28p. Although annotated as dispensable in the Stanford Yeast Genome Database (http://www.yeastgenome.org/cgi-bin/locus.fpl?locus=kre28), we find *KRE28* to be essential for vegetative growth. Moreover, based on medium-level sequence identity (which is typical of kinetochore proteins [46]), co-purifications in chicken cells (Fukagawa T., personal communication) and two-hybrid interactions between hSPC105 and Zwint-1 in human cells [15], we speculate that *KRE28* is probably orthologous to the metazoan kinetochore protein Zwint-1. Spc105p purified from yeast binds to MTs with an affinity similar to that of its *C. elegans* ortholog KNL-1 (∼1 µM) [27]. It is presently unclear whether Kre28p enhances the affinity of Spc105p for MTs. The low Kd value of Spc105p implies significantly less avid MT binding than the yeast Ndc80 complex (0.2–0.8 µM [6]). Indeed, the latter plays a critical role in MT attachment of budding yeast, *C. elegans* and human kinetochores [27], [40], [41], [44]. In *C. elegans*, it has been suggested that weak MT binding by KNL-1 serves to enhance binding by the Ndc80 complex via formation of a multivalent MT attachment site [27]. Perhaps this is also true of budding yeast Spc105p. However, it is not yet possible to determine whether a requirement for yeast Spc105p/Kre28p in efficient bi-orientation of sister kinetochores involves the intrinsic MT-binding activity of Spc105p/Kre28p or the role the complex plays in recruiting kMAPS and kinesins to the outer kinetochore [47].

**Figure 7:**
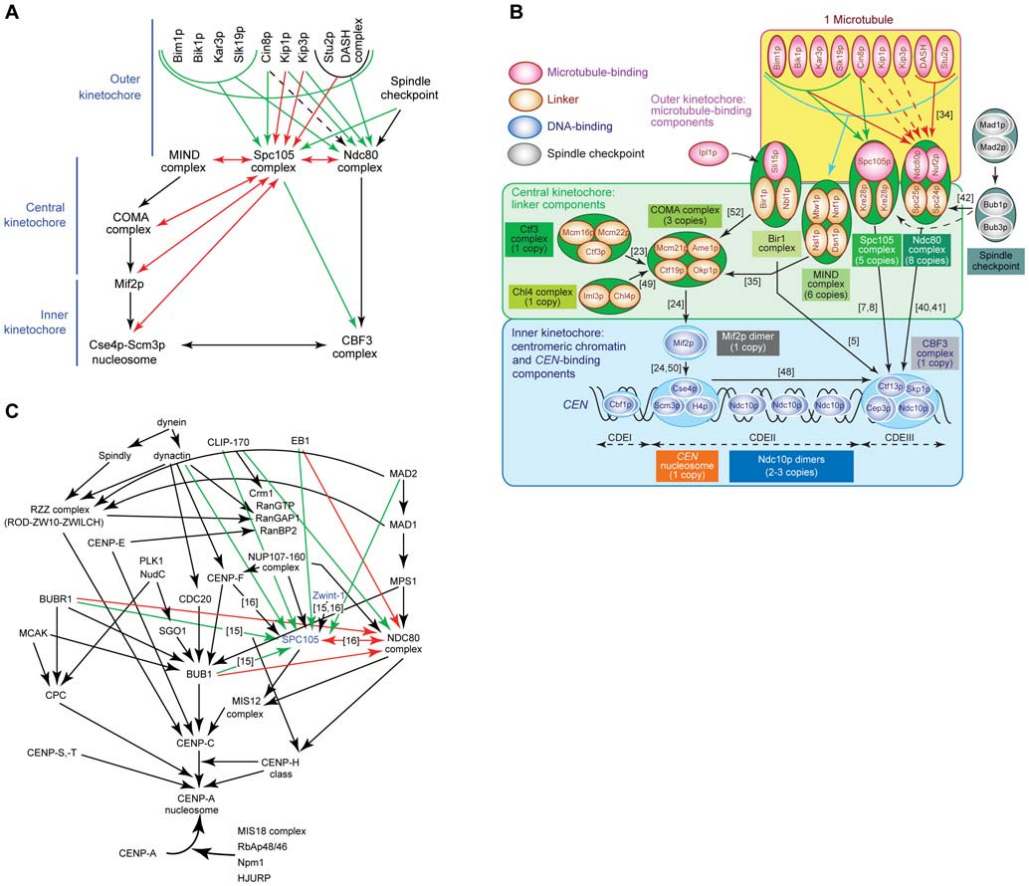
Hierarchical assembly of budding yeast and metazoan kinetochores. **(*A*)** *CEN*-protein recruitments requiring a functional yeast Spc105 complex, as shown in this study, are depicted with a green arrow. *CEN*-protein recruitments that do not require the Spc105 complex, as shown in this study, are depicted with a red arrow. Black arrows point to dependencies reported in the literature (for references, see panel *B*). **(*B*)** Diagram summarizing yeast kinetochore assembly on centromeres based on findings made in this study and those published in the literature ([5], [7], [8], [23], [24], [34], [35], [40], [41], [44], [48]–[50], [52]). Proteins colored pink bind to MTs, proteins colored blue bind to *CEN* DNA, proteins colored yellow constitute linker components, whereas spindle checkpoint proteins are colored grey. Encircled proteins form a stable kinetochore complex. The copy numbers of proteins and complexes were obtained from [4]. **(*C*)** Diagram summarizing vertebrate kinetochore assembly on centromeres [3]. Dependencies of kinetochore proteins on hSPC105 or hNDC80 for their localization to centromeres, as revealed in this study, are shown by green arrows. Recruitment independencies, as revealed in this study, are shown by red arrows.

Budding yeast kinetochores are hierarchical structures in which association of all known kinetochore proteins to *CEN* DNA requires prior binding by CBF3 to CDEIII [1]–[3]. CBF3 has orthologs only in a set of closely related budding yeast species but CBF3-CDEIII binding initiates assembly of a specialized domain of centromeric chromatin that is highly conserved throughout evolution [1]–[3]. A number of labs have previously shown that the heterotetrameric MIND and Ndc80 complexes require CBF3 for kinetochore association but not each other [7], [24], [35]. In the present study we find that the MIND and Ndc80 complexes are in turn required for kinetochore association of kMAPs and kinesins. Spc105p/Kre28p has a similar linker function to the MIND and Ndc80 complexes as it depends on CBF3, but not on the MIND or Ndc80 complexes for *CEN* binding. Moreover, Spc105p/Kre28p is required for *CEN*-association by the motors Kar3p and Cin8p, and the kMAPs Bik1p, Bim1p, and Slk19p. Thus Spc105p/Kre28p has the key properties of a linker complex. We had previously suggested that COMA might be a fourth such complex, being recruited to *CEN*-bound CBF3 independently of the MIND and Ndc80 complexes [7], but now conclude that COMA is in fact required for MIND-*CEN* binding as reported by Scharfenberger and colleagues [35]. The hydrodynamic properties of Spc105p/Kre28p show it to be elongated and similar in size and shape to the MIND and Ndc80 complexes [7]. These data, in combination with patterns of interdependency established previously [5], [7], [8], [23], [24], [34], [35], [40], [41], [43], [48]–[52] make it possible to create a functional model of yeast kinetochores that encompasses ∼70 subunits (Fig. 7A,B). A key feature of this model is the elongated core of linker complexes (light green in Fig. 7B) having intrinsic MT-binding activity and the ability to nucleate the assembly of a multi-component outer kinetochore (yellow in Fig. 7B). The precise relationship between linkers and components of the outer kinetochore remains unclear. However, the studies in this paper clearly show that no simple one-to-one correspondence exists; in nearly all cases multiple linkers are required for kinetochore recruitment of a single kMAP or motor (Fig. 7A,B).

How similar are the linker components of higher eukaryotes to their budding yeast counterparts? In humans we find that kinetochore binding by the hNDC80 complex occurs independently of the hSPC105 complex, confirming previous reports [15], [16]. Reversely, we also find that hSPC105 does not require hNDC80, confirming that both complexes -similar to budding yeast- reside in parallel within human kinetochores [16]. However, the human MIS12 complex (ortholog of the yeast Mtw1/MIND complex) is required for kinetochore association by the hSPC105 [16] and hNDC80 complexes [53], a clear difference with budding yeast in which the three complexes exist in parallel. In *C. elegans*, the MIS12 complex is required for KNL-1/Spc105 binding and this in turn leads to recruitment of Ndc80, creating what appears to be a linear pathway of kinetochore assembly [11], [12]. Finally, in *Drosophila*, the Spc105 and Mis12 complexes are involved in recruiting the Ndc80 complex. However, the Scp105 and Mis12 complexes depend on each other for recruitment to centromeres [17], [54]. Overall, these data suggest that MIND, Ndc80 and Spc105 linker components are in intimate association in multiple organisms, but that the details of their interaction differ. These differences are also apparent with respect to function: whereas inactivation of yeast Spc105p affects sister kinetochore bi-orientation, MT binding *per se* is not abolished. In humans and *Drosophila*, in contrast, Spc105 inactivation completely abolishes kinetochore-MT attachment (this study; [15], [17]). A striking feature of detached, hSPC105-depleted kinetochores is that they contain normal levels of hNDC80. hNDC80 is widely described as the key complex in human kinetochore-MT binding, but our results and those from others [15] clearly show that that the hNDC80 complex cannot mediate MT attachment in the absence of hSPC105 and the proteins it recruits to kinetochores.

In all eukaryotes examined to date, the binding of the spindle checkpoint to kinetochores depends on the Ndc80 complex [40], [41], [43], [55]. In budding yeast, we show involvement of Spc105p/Kre28p in checkpoint activity as we observed reduced levels of the Mad2 and Bub1 checkpoint proteins on unattached kinetochores in *spc105* mutants, resulting in a transient but not a normally sustained checkpoint-mediated delay in the face of MT depolymerization. Our data combined with those by Kiyomitsu et al. [15] show that next to the hNDC80 complex, also hSPC105 plays an essential role in human spindle checkpoint function. Importantly, though, we find that hSPC05 recruits hBUB1 and hBUBR1 while the hNDC80 complex does not. More surprising is the recent finding that *Drosophila spc105* mutants are checkpoint-proficient despite the absence of kinetochore-localized Ndc80 complex [17]. Perhaps this reflects the fundamental difference in assembly hierarchy of kinetochores in *Drosophila* versus that in other organisms. Alternatively, working out the role of the Ndc80 complex in checkpoint control is challenging: for instance, only when levels of hNDC80 complex subunits were depleted below ∼1% of wild-type level was the human spindle checkpoint inactivated [56].

In summary, key aspects of Spc105 function are conserved from budding yeast to human cells - including a linker function in recruitment of certain kMAPs and motors. Involvement in MT-binding and spindle checkpoint recruitment also appears to have been conserved from yeast to humans although hSPC105 is more prominent in both activities (e.g., recruitment versus maintenance of the spindle checkpoint). The functional consequences of Spc105 inactivation differ among species presumably reflecting differences in the hierarchy of kinetochore assembly and in the roles played by kinetochores in chromosome segregation: *de novo* MT capture, for example, is more important in human cells than in budding yeast, in which kinetochores remain MT-bound throughout the cell cycle.

## Materials and Methods

### Yeast strains and growth conditions

All strains were derived from W303 (Table S1) via standard methods for epitope tagging and gene deletion. For cell cycle analysis, wild-type, *spc105^ts^* and *mad2α* strains were grown in YPD at 24°C and synchronized with α-factor (2 h, 10 mg/ml). The cultures were then shifted to 37°C (1 h), and 5 mg/ml of α-factor was added to hold the cells in G1 while Spc105-4p^ts^ or Spc105-15p^ts^ were inactivated at 37°C for 2 h. Cells were then released into fresh YPD medium at 37°C. To establish viability curves, samples taken after release from α-factor were diluted and plated on YPD agar. After 4 days of incubation at 24°C, the number of colonies were counted. The cytokinesis defect of *spc105* mutants was scored following zymolyase treatment [57], and by tracking septin ring duplication (Cdc3p-GFP [9]). Microscopy was carried out with a Deltavision deconvolution microscope.

### Chromatin immunoprecipitation

Chromatin immunoprecipitation (ChIP) assays were performed as described [7]. In anti-Cep3p ChIP assays, polyclonal anti-Cep3p antibody was used (Sorger lab); for ChIP of GFP-tagged proteins, “Living Colors Full-Length A.V. Polyclonal Antibody” (BD Biosciences) was used; for ChIP of protein-A tagged proteins, IgG Sepharose CL-4B beads were used (Amersham Biosciences).

Oligomers 5′-GCGCAAGCTTGCAAAAGGTCACATG-3′ and 5′-CGAATTCATTTTGGCCGCTCCTAGGTA-3′ were used to amplify *CEN4*. Oligomers 5′-GGCTGTGGTTTCAGGGTCCATAAAGC-3′ and 5′-CTGGGCAATTTCATGTTTCTTCAACACC-3′ were used to amplify *URA3* (negative control). PCRs were performed with Taq Polymerase (New England Biolabs).

### Biochemical analysis

Protein immunoprecipitations (IPs) were performed as follows: whole cell extracts from yeast strains expressing epitope-tagged proteins were prepared with glass beads (FastPrep 120, QBiogene) and 1 mg of total protein was incubated with mouse anti-HA antibody (MMS-101R, CRP Inc.) or mouse anti-Myc antibody (9E10, abCAM) in a 1∶50 (v∶v) ratio. These antibodies (1∶500) were also used for western blotting of the IPs. Gel filtration (HiPrep 16/60 Sephacryl S-500 HR, Amersham Biosciences), 10–40% glycerol-gradient velocity ultracentrifugation (Biocomp Instruments), and hydrodynamic calculations were done as described [7]. Standards used in the ultracentrifugation analyses (Fig. 1A) are “R”: bovine pancreas ribonuclease A, Svedberg coefficient S = 1.78×10^−13^ s; “Ch”: bovine pancreas chymotrypsinogen A, S = 2.58×10^−13^ s; “O”: hen egg ovalbumin, S = 3.55×10^−13^ s; “B”: bovine serum albumin, S = 4.22×10^−13^ s; “A”: rabbit muscle aldolase, S = 7.45×10^−13^ s; “Ca”: bovine liver catalase, S = 11.3×10^−13^ s. Standards used in the gel filtration analyses (Fig. 1A) are “T”: bovine thyroid thyroglobulin, diffusion coefficient D = 2.49×10^−7 ^cm^2^s^−1^; “F”: horse spleen ferritin, D = 3.61×10^−7^ cm^2^s^−1^; “Ca”: bovine liver catalase, D = 4.1×10^−7^ cm^2^s^−1^; “B”: bovine serum albumin, D = 6.8×10^−7^ cm^2^s^−1^ (GE Healthcare).

Purification and MT-binding analysis of Spc105p were performed as follows: after 12 h of growth of PDWY1264 in YP galactose (2%) medium, GST-Spc105p was purified from whole cell extracts by incubation with glutathione beads, followed by release with Precision protease (GE Healthcare Life Sciences). 1 µM of Spc105p was then incubated for 10 min (24°C) with 0–10 mM of taxol-stabilized bovine MTs (Cytoskeleton). Following ultracentrifugation (10 min; 90,000 rpm), the presence of Spc105p in supernatant and MT-pellet fractions was determined via SDS-PAGE and quantified with ImageJ. The Kd was calculated with Prism (GraphPad).

### HeLa cell experiments

HeLa cells were grown in media containing 10% fetal bovine serum. Nocodazole (1 mM) was added freshly. Mitotic cells were studied following release from G1/S arrest by double thymidine treatment (2 mM). Polyclonal antibodies against hSPC105 peptide SRDEENKKSHNGAETTS were generated in Guinea pig. Immunofluorescence analysis with CREST serum or antibodies against hSPC105, hNDC80, hMAD2, hBUB1, hBUBR1, ROD, EB1, p150^Glued^, CLIP-170, or β-tubulin were performed as described [56], [58]. For siRNA-mediated depletion, conditions were used as described [56]. For hSPC105-siRNA, oligomers HSS125942, HSS125943 and HSS125944 (Invitrogen) were used. For hNDC80 siRNA, we used the oligomers described previously [56].

## Supporting Information

Figure S1 ChIP-based *CEN*-recruitment analysis of kinetochore proteins in various yeast kinetochore mutants. The data (summarized in Table 1 and Fig. 7A,B) come from experiments performed in triplicate and were related to the % of Cep3p that was ChIPed in parallel (positive control). For strains, see Table S1.
(0.94 MB TIF)

Figure S2 Budding yeast mutants lacking Spc105p activity do not arrest in mitosis and are compromised in cytokinesis. (A) *spc105* mutants released from G1 arrest with α-factor do not arrest at non-permissive temperature (37°C) but gradually lose viability. (B) Loss of Spc105p activity compromises cytokinesis as illustrated by the inability of the cells to separate following treatment with cell wall digesting enzyme zymolyase [52]. (D) *spc105* mutants are defective in cytokinesis as illustrated by their inability to duplicate the septin ring (Cdc3-GFP [9]).
(1.87 MB TIF)

Figure S3 Loss of Spc105p activity abrogates spindle checkpoint function. FACS data of the experiment shown in Figure 4A.
(0.39 MB TIF)

Figure S4 The antibody raised against hSPC105 recognizes hSPC105 in western blotting and indirect immunofluorescence experiments. (A) Western blot of HeLa cell lysates probed with an anti-hSPC105 antibody (hSPC105: ∼300 kDa). (B) Immunofluorescence (IF) imaging of mitotic HeLa cells stained with CREST sera (inner kinetochore, red), anti-hSPC105 antibody (green), and DAPI (DNA, blue). Scale Bar: 10 µm. Insets are 13× magnifications. (C) Anti-hSPC105 and anti-β-tubulin western blots of HeLa cell lysates treated with anti-hSPC105 or control siRNA oligos.
(0.47 MB TIF)

Table S1 Yeast strains used in this study.
(0.13 MB DOC)
