# Supplementary figures and images for: Roles for the Conserved Spc105p/Kre28p Complex in Kinetochore-Microtubule Binding and the Spindle Assembly Checkpoint
Source: PLoS One. 2009 Oct 28;4(10):e7640. doi: 10.1371/journal.pone.0007640 (PMC2764089; doi:10.1371/journal.pone.0007640)

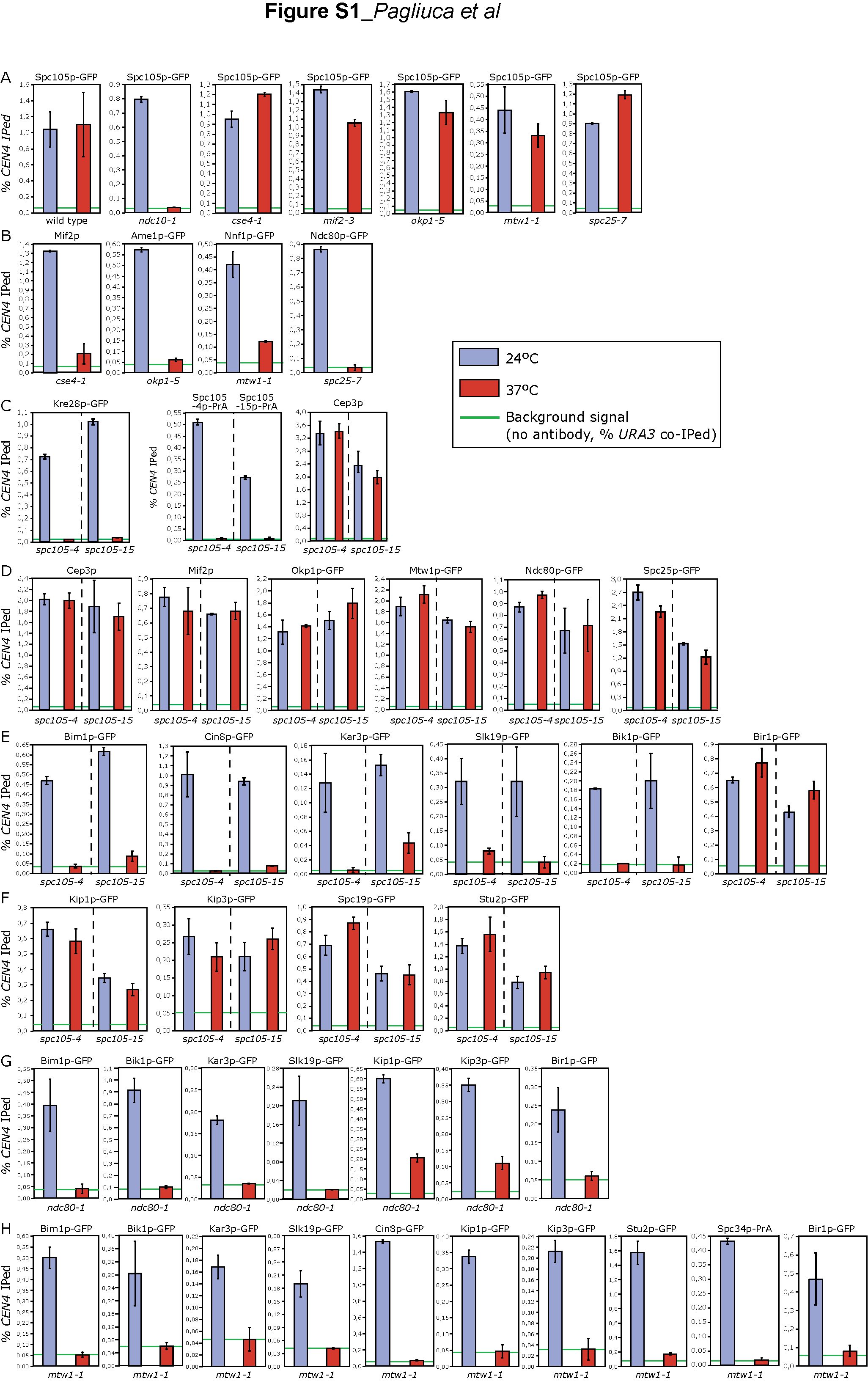

Supplement: Figure S1 — ChIP-based CEN-recruitment analysis of kinetochore proteins in various yeast kinetochore mutants. The data (summarized in Table 1 and Fig. 7A,B) come from experiments performed in triplicate and were related to the % of Cep3p that was ChIPed in parallel (positive control). For strains, see Table S1. (0.94 MB TIF) [file pone.0007640.s001.tif]

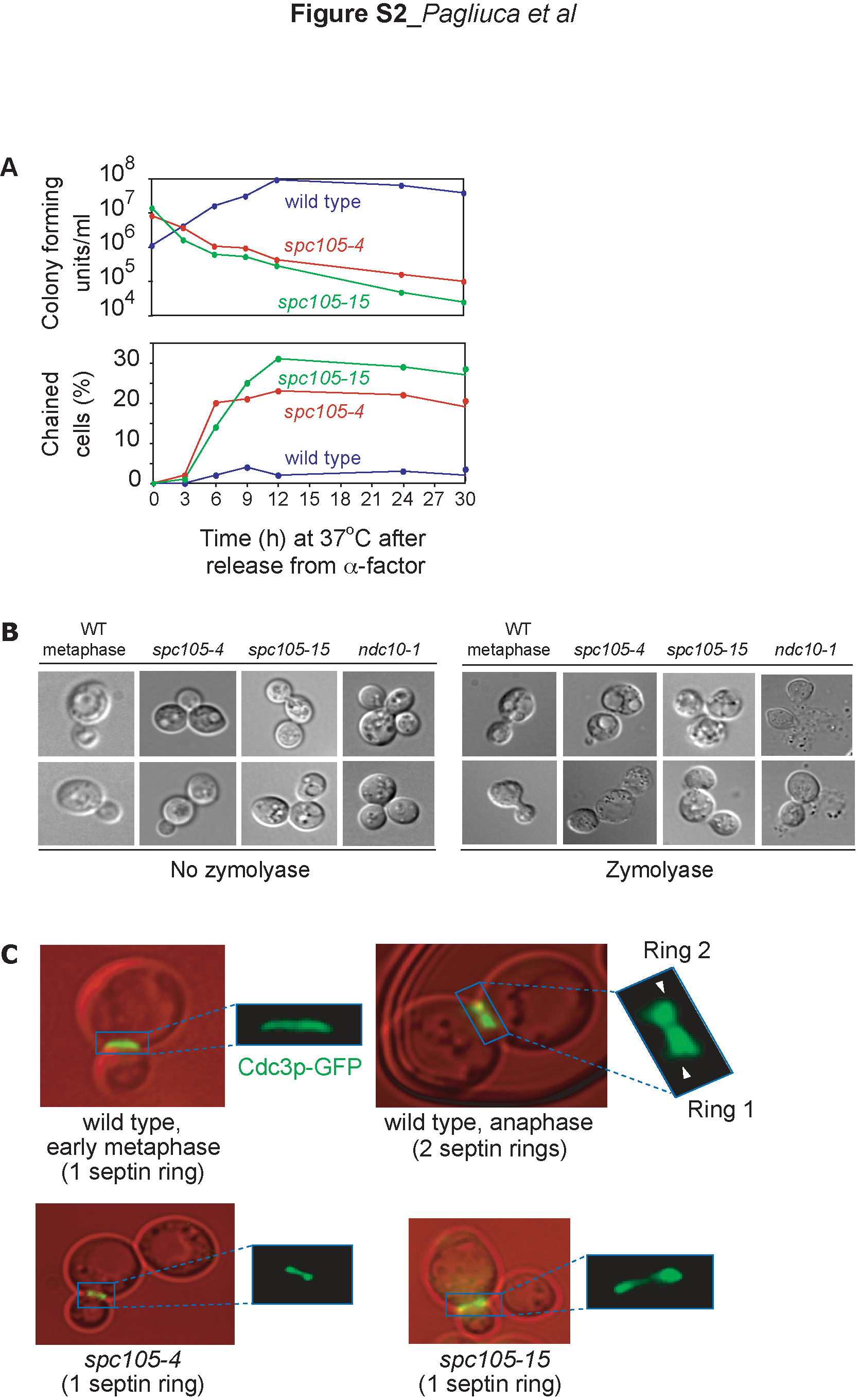

Supplement: Figure S2 — Budding yeast mutants lacking Spc105p activity do not arrest in mitosis and are compromised in cytokinesis. (A) spc105 mutants released from G1 arrest with α-factor do not arrest at non-permissive temperature (37°C) but gradually lose viability. (B) Loss of Spc105p activity compromises cytokinesis as illustrated by the inability of the cells to separate following treatment with cell wall digesting enzyme zymolyase [52]. (D) spc105 mutants are defective in cytokinesis as illustrated by their inability to duplicate the septin ring (Cdc3-GFP [9]). (1.87 MB TIF) [file pone.0007640.s002.tif]

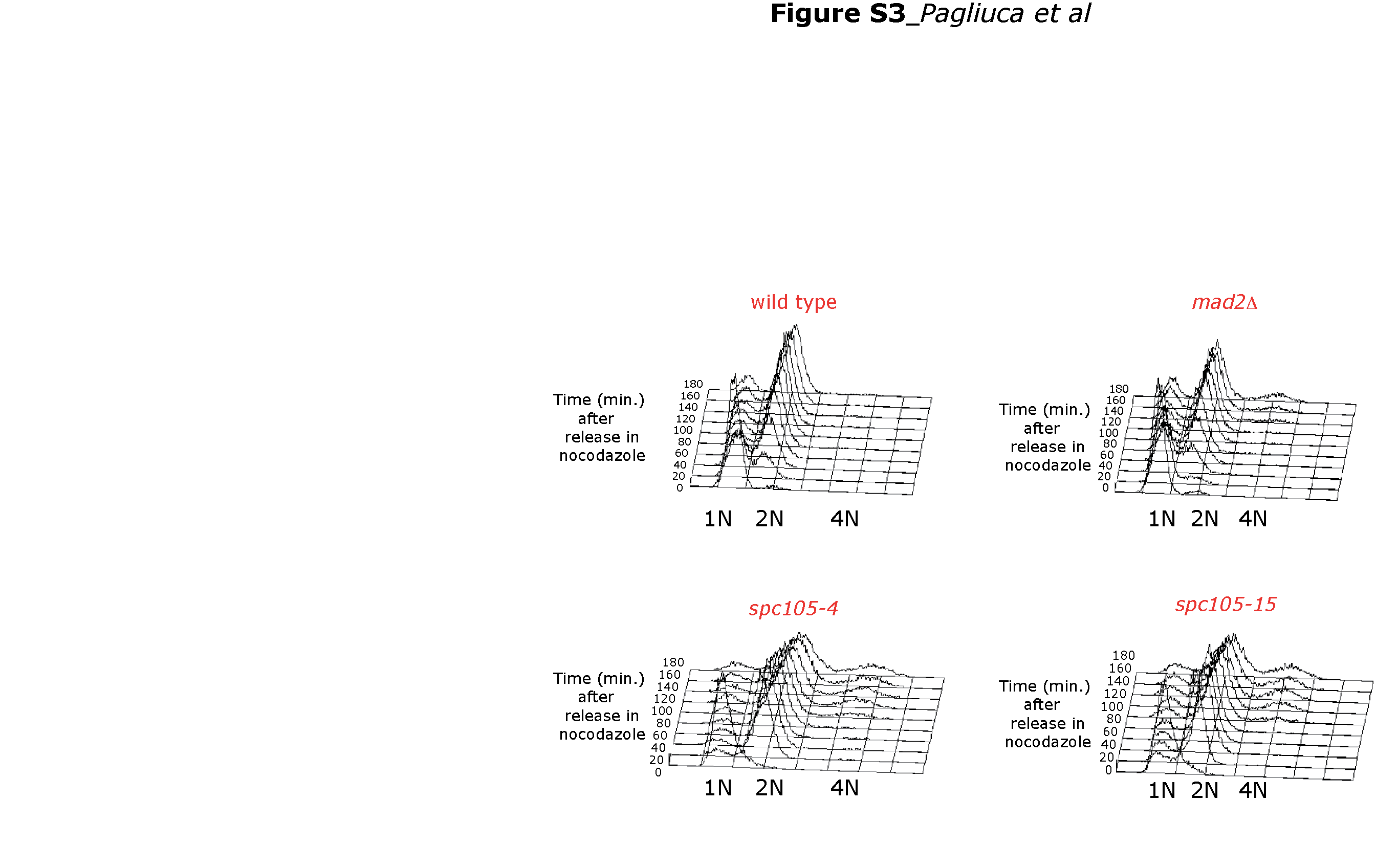

Supplement: Figure S3 — Loss of Spc105p activity abrogates spindle checkpoint function. FACS data of the experiment shown in Figure 4A. (0.39 MB TIF) [file pone.0007640.s003.tif]

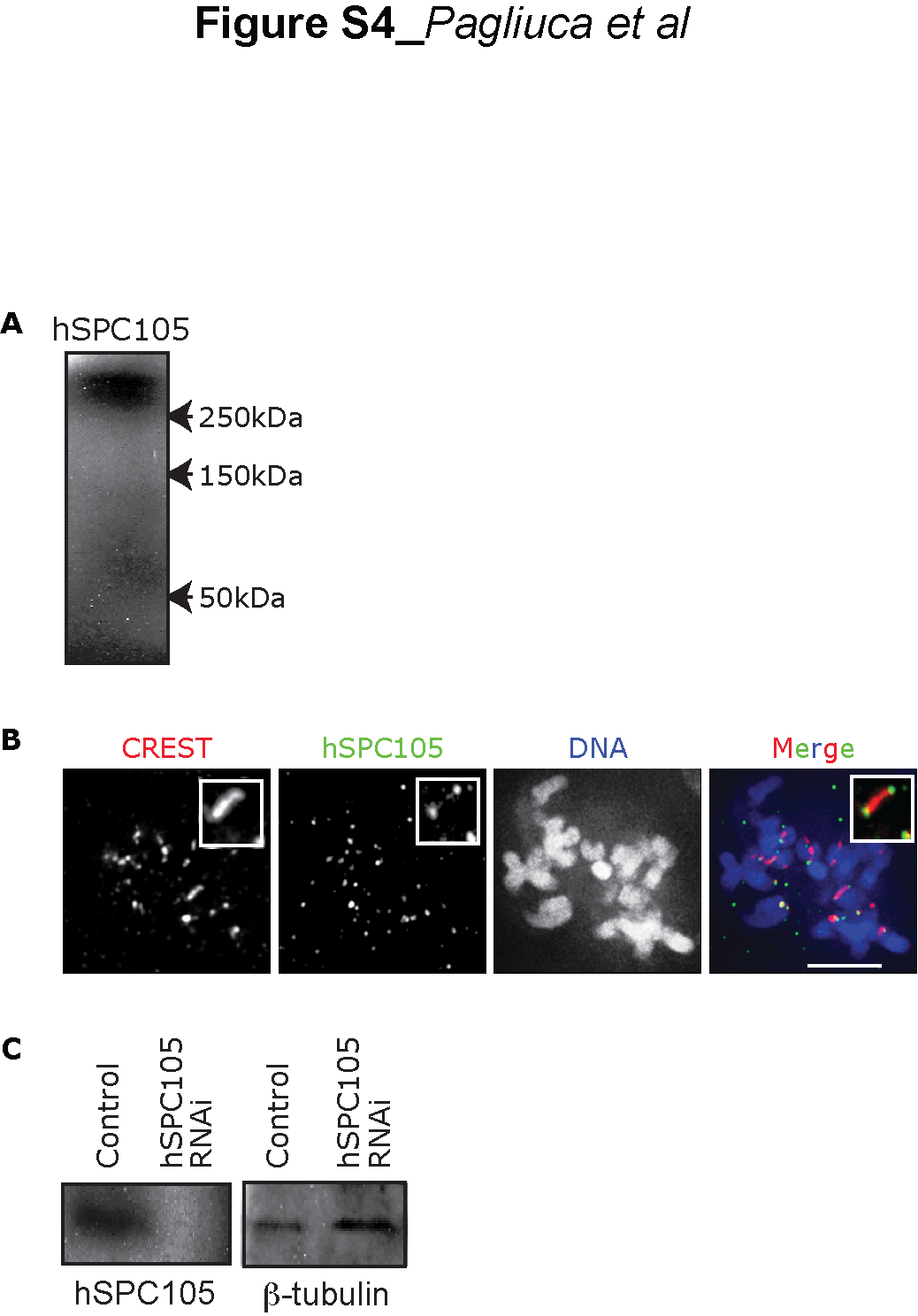

Supplement: Figure S4 — The antibody raised against hSPC105 recognizes hSPC105 in western blotting and indirect immunofluorescence experiments. (A) Western blot of HeLa cell lysates probed with an anti-hSPC105 antibody (hSPC105: ∼300 kDa). (B) Immunofluorescence (IF) imaging of mitotic HeLa cells stained with CREST sera (inner kinetochore, red), anti-hSPC105 antibody (green), and DAPI (DNA, blue). Scale Bar: 10 µm. Insets are 13× magnifications. (C) Anti-hSPC105 and anti-β-tubulin western blots of HeLa cell lysates treated with anti-hSPC105 or control siRNA oligos. (0.47 MB TIF) [file pone.0007640.s004.tif]
