# Supplementary material for: Roles for the Conserved Spc105p/Kre28p Complex in Kinetochore-Microtubule Binding and the Spindle Assembly Checkpoint
Source: PLoS One. 2009 Oct 28;4(10):e7640. doi: 10.1371/journal.pone.0007640 (PMC2764089; doi:10.1371/journal.pone.0007640)
Supplement: Table S1 — Yeast strains used in this study. (0.13 MB DOC) [file pone.0007640.s005.doc]

**Table S1.** **Yeast strains used in this study**

| Strain | Genotype | Reference |
| --- | --- | --- |
| PDWY105 | *ndc80-1* | 1 |
| PDWY110 | *mtw1-1* | 2 |
| PDWY111 | *ndc10-1* | 3 |
| PDWY128 | *okp1-5* | 4 |
| PDWY411 | *mif2-3* | 5 |
| PDWY148 | *cse4-1* | 6 |
| PDWY230 | *spc105-4* | 7 |
| PDWY231 | *spc105-15* | 7 |
| PDWY367 | *spc25-7* | 8 |
| PDWY077 | *okp1-5* *AME1::TAP-URA3* | 9 |
| PDWY394 | *spc25-7* *NDC80::GFP-URA3* | 10 |
| PDWY405 | *spc25-7* *BUB1::GFP-URA3* | 10 |
| PDWY406 | *spc25-7* *MAD2::GFP-URA3* | 10 |
| PDWY350 | *PDS1::13MYC-KanMX6* | O. Cohen-Fix |
| PDWY387 | *mad2D::KanMX6* | This study |
| PDWY136 | *mtw1-1* *SPC34::PrA-URA3* | This study |
| PDWY155 | *SPC105::TEV-PrA-KanMX6* | This study |
| PDWY156 | *ndc10-1* *SPC105::GFP-TRP1* | This study |
| PDWY157 | *mtw1-1* *SPC105::GFP-HIS3MX6* | This study |
| PDWY158 | *cse4-1* *SPC105::GFP-HIS3MX6* | This study |
| PDWY159 | *ndc80-1* *SPC105::GFP-HIS3MX6* | This study |
| PDWY160 | *okp1-5* *SPC105::GFP-HIS3MX6* | This study |
| PDWY201 | *YDR532c::TEV-PrA-KanMX6* | This study |
| PDWY237 | *spc105-4* *YDR532c::GFP-His3MX6* | This study |
| PDWY238 | *spc105-4* *YDR532c::TEV-PrA-KanMX6* | This study |
| PDWY240 | *spc105-15* *YDR532c::TEV-PrA-KanMX6* | This study |
| PDWY242 | *spc105-15* *YDR532c::GFP-His3MX6* | This study |
| PDWY273 | *KRE28::3HA-His3MX6 KRE28::13MYC-KanMX6* | This study |
| PDWY274 | *KRE28::3HA-His3MX6/KRE28*  *SPC105::13MYC-KanMX6/SPC105* | This study |
| PDWY275 | *SPC105::TEV-PrA-7HIS-His3MX6* | This study |
| PDWY301 | *SPC105-4::TEV-PrA-KanMX6* | This study |
| PDWY303 | *SPC105-15::TEV-PrA-KanMX6* | This study |
| PDWY313 | *spc105-4* *NDC80::GFP-URA3* | This study |
| PDWY315 | *spc105-15* *NDC80::GFP-URA3* | This study |
| PDWY332 | *spc105-4* *MAD2::GFP-URA3 SPC42::CFP-TRP1* | This study |

| PDWY334 | *spc105-4* *BUB1::GFP-URA3 SPC42::CFP-TRP1* | This study |
| --- | --- | --- |
| PDWY338 | *spc105-15* *MAD2::GFP-URA3 SPC42::CFP-TRP1* | This study |
| PDWY340 | *spc105-15* *BUB1::GFP-URA3 SPC42::CFP-TRP1* | This study |
| PDWY355 | *spc105-15* *PDS1::13MYC-KanMX6* | This study |
| PDWY357 | *spc105-4* *PDS1::13MYC-KanMX6* | This study |
| PDWY359 | *spc105-4* *OKP1::GFP-His3MX6* | This study |
| PDWY361 | *spc105-15* *OKP1::GFP-His3MX6* | This study |
| PDWY363 | *spc105-4* *MTW1::GFP-His3MX6* | This study |
| PDWY365 | *spc105-15* *MTW1::GFP-His3MX6* | This study |
| PDWY373 | *spc25-7* *SPC105::GFP-TRP1* | This study |
| PDWY379 | *spc105-4* *SPC25::GFP-His3MX6* | This study |
| PDWY381 | *spc105-15* *SPC25::GFP-His3MX6* | This study |
| PDWY397 | *mad2D::URA3* *PDS1::13MYC-KanMX6* | This study |
| PDWY399 | *ndc80-1* *NUF2::GFP-TRP1* | This study |
| PDWY401 | *spc105-4* *SPC19::GFP-URA3* | This study |
| PDWY403 | *spc105-15* *SPC19::GFP-URA3* | This study |
| PDWY409 | *spc105-4 BIM1::GFP-TRP1* | This study |
| PDWY410 | *spc105-15 BIM1::GFP-TRP1* | This study |
| PDWY412 | *mif2-3 SPC105::GFP-KanMX6* | This study |
| PDWY414 | *mtw1-1* *BIM1::GFP-His3MX6* | This study |
| PDWY418 | *ndc80-1* *BIM1::GFP-His3MX6* | This study |
| PDWY425 | *spc105-15* *CIN8::GFP-His3MX6* | This study |
| PDWY429 | *spc105-4* *CIN8::GFP-His3MX6* | This study |
| PDWY430 | *mif2-3* *BIM1::GFP-KanMX6* | This study |
| PDWY432 | *mtw1-1* *CIN8::GFP-URA3* | This study |
| PDWY434 | *mtw1-1* *STU2::GFP-URA3* | This study |
| PDWY436 | *spc105-4* *STU2::GFP-URA3* | This study |
| PDWY438 | *spc105-15* *STU2::GFP-URA3* | This study |
| PDWY440 | *spc105-4* *KIP1::GFP-URA3* | This study |
| PDWY442 | *spc105-15* *KIP1::GFP-URA3* | This study |
| PDWY444 | *spc105-4* *KIP3::GFP-URA3* | This study |
| PDWY446 | *spc105-15* *KIP3::GFP-URA3* | This study |
| PDWY448 | *mtw1-1* *KIP1::GFP-URA3* | This study |
| PDWY450 | *mtw1-1* *KIP3::GFP-URA3* | This study |
| PDWY452 | *spc105-15* *KAR3:: GFP-His3MX6* | This study |
| PDWY457 | *ndc80-1 KIP1::GFP-URA3* | This study |
| PDWY459 | *ndc80-1 KIP3::GFP-URA3* | This study |
| PDWY461 | *spc105-4 KAR3::GFP-HIS3* | This study |
| PDWY463 | *ndc80-1 KAR3::GFP-HIS3* | This study |
| PDWY465 | *mtw1-1 KAR3::GFP-HIS3* | This study |
| PDWY507 | *mtw1-1 SLK19::GFP-URA3* | This study |
| PDWY509 | *ndc80-1 SLK19::GFP-URA3* | This study |
| PDWY511 | *spc105-4 SLK19::GFP-URA3* | This study |
| PDWY513 | *spc105-15 SLK19::GFP-URA3* | This study |
| PDWY527 | *ndc80-1 BIK1::GFP-His3MX6* | This study |
| PDWY529 | *mtw1-1 BIK1::GFP-His3MX6* | This study |
| PDWY530 | *spc105-4 BIK1::GFP-His3MX6* | This study |
| PDWY532 | *spc105-4::PrA-KanMX4, CENIV::TetR-GFP-LEU2, TETO::URA3, SPC42::GFP-TRP1* | This study |
| PDWY533 | *spc105-15 BIK1::GFP-His3MX6* | This study |
| PDWY571 | *spc105-15 pCDC3::GFP-CEN6-URA3* | This study |
| PDWY573 | *spc105-4 pCDC3::GFP-CEN6-URA3* | This study |
| PDWY575 | *pCDC3::GFP-CEN6-URA3* | This study |
| PDWY1264 | *mob1D::KanMX4, pep4D::LEU2, pGST::SPC105-2m-URA3-MOB1* | This study |

All strains are MATa, except for PDWY273 and PDWY274, which are diploid.

References

1. Wigge PA, Kilmartin JV (2001) The Ndc80p complex from *Saccharomyces cerevisiae* contains conserved centromere components and has a function in chromosome segregation. J Cell Biol 152: 349-360.

2. Goshima G, Yanagida M (2000) Establishing biorientation occurs with precocious separation of the sister kinetochores, but not the arms, in the early spindle of budding yeast. Cell 100: 619-633.

3. Goh PY, Kilmartin JV (1993) NDC10: a gene involved in chromosome segregation in *Saccharomyces cerevisiae.* J Cell Biol 121: 503-512.

4. Ortiz J, Stemmann O, Rank S, Lechner J (1999) A putative protein complex consisting of Ctf19, Mcm21, and Okp1 represents a missing link in the budding yeast kinetochore. Genes Dev 13: 1140-1155.

5. [Brown MT](http://www.ncbi.nlm.nih.gov/entrez/query.fcgi?db=pubmed&cmd=Search&itool=pubmed_AbstractPlus&term="Brown+MT"%5BAuthor%5D), [Goetsch L](http://www.ncbi.nlm.nih.gov/entrez/query.fcgi?db=pubmed&cmd=Search&itool=pubmed_AbstractPlus&term="Goetsch+L"%5BAuthor%5D), [Hartwell LH](http://www.ncbi.nlm.nih.gov/entrez/query.fcgi?db=pubmed&cmd=Search&itool=pubmed_AbstractPlus&term="Hartwell+LH"%5BAuthor%5D) (1993) MIF2 is required for mitotic spindle integrity during anaphase spindle elongation in *Saccharomyces* *cerevisiae*. [J Cell Biol](javascript:AL_get(this, 'jour', 'J Cell Biol.');) 123: 387-403.

6. Stoler S, Keith KC, Curnick KE, Fitzgerald-Hayes MA (1995) Mutation in CSE4, an essential gene encoding a novel chromatin-associated protein in yeast, causes chromosome nondisjunction and cell cycle arrest at mitosis. Genes Dev 9: 573-586.

7. Nekrasov VS, Smith MA, Peak-Chew S, Kilmartin JV (2003) Interactions between centromere complexes in *Saccharomyces cerevisiae*. Mol Biol Cell 14: 4931-4946.

8. Janke C, Ortiz J, Lechner J, Shevchenko A, Shevchenko A, Magiera MM, Schramm C, Schiebel E (2001) The budding yeast proteins Spc24p and Spc25p interact with Ndc80p and Nuf2p at the kinetochore and are important for kinetochore clustering and checkpoint control. EMBO J 20: 777-791.

9. De Wulf P, McAinsh AD, Sorger PK (2003) Hierarchical assembly of the budding yeast kinetochore from multiple subcomplexes. Genes Dev 17: 2902-2921.

10. Gillett ES, Espelin CW, Sorger PK (2004) Spindle checkpoint proteins and chromosome-microtubule attachment in budding yeast. J Cell Biol 164: 535-546.
